# Effectiveness of isopropyl myristate/cyclomethicone D5 solution of removing cuticular hydrocarbons from human head lice (*Pediculus humanus capitis*)

**DOI:** 10.1186/1471-5945-12-15

**Published:** 2012-09-03

**Authors:** Eric Barnett, Kathleen G Palma, Bert Clayton, Timothy Ballard

**Affiliations:** 1 Piedmont Pharmaceuticals, Greensboro, NC, USA; 2 En-Cas Analytical Laboratories, Winston-Salem, NC, USA

**Keywords:** Non-pesticidal, Head lice, Therapy, Treatment, Resistance, Permethrin, Pyrethroid, Isopropyl myristate, Cyclomethicone D5, Decamethylcyclopentasiloxane

## Abstract

**Background:**

In the treatment of human head lice infestation, healthcare providers are increasingly concerned about lice becoming resistant to existing pesticide treatments. Traditional pesticides, used to control these pests, have a neurological mechanism of action. This publication describes a topical solution with a non-traditional mechanism of action, based on physical disruption of the wax layer that covers the cuticle of the louse exoskeleton. This topical solution has been shown clinically to cure 82% of patients with only a 10-minute treatment time, repeated once after 7 days. All insects, including human head lice, have a wax-covered exoskeleton. This wax, composed of hydrocarbons, provides the insect with protection against water loss and is therefore critical to its survival. When the protective wax is disrupted, water loss becomes uncontrollable and irreversible, leading to dehydration and death. A specific pattern of hydrocarbons has been found in all of the head louse cuticular wax studied. Iso-octane effectively removes these hydrocarbons from human head lice’s cuticular wax.

**Methods:**

A method of head louse cuticle wax extraction and analysis by gas chromatography was developed. Human head lice (*Pediculus humanus capitis*) were collected from infested patients and subjected to any of three extraction solvents comprising either the test product or one of two solvents introduced as controls. A gas chromatograph equipped with a flame ionization detector (GC/FID) was used to determine the presence of hydrocarbons in the three head lice extracts.

**Results:**

In the study reported herein, the test product isopropyl myristate/cyclomethicone D5 (IPM/D5) was shown to perform comparably with iso-octane, effectively extracting the target hydrocarbons from the cuticular wax that coats the human head louse exoskeleton.

**Conclusions:**

Disruption of the integrity of the insect cuticle by removal of specific hydrocarbons found in the cuticular wax appears to offer a mechanism for killing lice without the likelihood of encountering genetic resistance.

## Background

### Head lice a global issue

Head lice infestation is a fairly common problem globally, with 6–12 million cases in children ages 3–11 reported annually in the US alone [1], and is typically observed in the school-aged population. Infestation is transmitted most commonly by physical head-to-head contact, but also by sharing hats, hairbrushes, headbands, or clothing.

### Insect exoskeleton-based cuticular hydrocarbons are universal

Head lice, like all insects, have a protective waxy covering on the epicuticle (outer layer of the cuticle on their exoskeletons) that acts as waterproofing for the insect. Therefore, without the wax, the insect is vulnerable to uncontrollable dehydration, and death. These cuticular lipids are composed of various hydrocarbons whose patterns vary by insect [2].

### A challenge to cure

Head lice infestations can be difficult to cure completely, and typically require multiple treatments. Some products may act by suffocation of the insects; these may require up to an 8 hour application time. However with additional changes made to the suffocant formulation the 8 hour treatment can be decreased substantially. Eggs (nits) are attached to the hair with protein ‘glue’ which makes the eggs difficult to remove. Even after treatment, viable eggs may hatch and young nymphs emerge. Isopropyl myristate (IPM) and cyclomethicone D5 do not have an effect on egg development. Only after the operculum (the opening the nymphal louse emerges from) begins to open (4–5 days) can the IPM and cyclomethicone D5 come in contact with the nymph inside. Once the nymph is exposed to the formulation, the nymph will die whether it has emerged from the egg or not.

### Pesticide therapies

Some medical practitioners recommend pesticide treatments containing permethrin, pyrethrin, or malathion. These therapies have been the first line of treatment for head lice since World War II. Today more interest is being placed on treatments that are alternatives to conventional pesticides with different modes of action. These new therapies are unlikely to allow development of resistance in the head louse population.

### Increasing resistance to pesticides

Of particular concern, is the growing body of evidence suggesting a global emergence in head lice of significant resistance to pesticide products containing permethrins and pyrethroids. Marcoux et al. reported that the widespread use of pyrethrins and pyrethroids have led to significant resistance across various countries including Canada, Argentina, Australia, the United Kingdom, the United States, Japan, Korea, and Israel. This resistance results in prolonged infestations, reinfestation and safety concerns resulting from overdosing with a treatment therapy. Resistance factors associated with insensitivity to DDT, pyrethrins and pyrethroids were found by Marcoux to be present in 97% of head lice collected from several Canadian cities [3].

### Chemistry of Isopropyl Myristate/Cyclomethicone D5 (IPM/D5)

Isopropyl myristate/cyclomethicone D5, is a topical formulation containing 50/50 w/w Isopropyl myristate/decamethylcyclopentasiloxane. Isopropyl myristate, with the chemical formula CH_3_(CH_2_)_12_COOCH (CH_3_)_2 _[4], is an ester of myristic acid, an essential fatty acid derived from palm kernel oil and isopropyl alcohol. IPM is used to dissolve lanolin and other oils, and is commonly used as a degreasant or softener in sunscreens, face creams and lipsticks. Due to its probable physical mechanism of action (dissolution of the waxy cuticular covering found on the louse exoskeleton), head louse resistance is not expected to develop against isopropyl myristate [3]. Decamethylcyclopentasiloxane (cyclomethicone D5), with the chemical formula *cyclo*-(Me_2_SiO)_5 _[5] is a spreading agent commonly used in cosmetics, that helps the isopropyl myristate to thoroughly coat the hair. Some adulticide activity has been noted in laboratory studies at concentrations higher than 50%. Cyclomethicone D5, which is used extensively as an excipient, can be found in some head lice treatments in concentrations as high as 96%.

### About this study

This study was conducted in an attempt to investigate the mechanism by which IPM/D5 is able to kill head lice after 10 minutes contact. It is well understood that insect dehydration can kill quickly, and it was expected that IPM/D5 was acting via a physical mechanism to disrupt the protective waxy coating on the louse exoskeleton. This study investigates this possible mechanism of action by specifically determining the removal efficiency of head louse cuticular hydrocarbons using a 50/50 w/w mixture of isopropyl myristate/cyclomethicone D5.

## Methods

A method of head louse cuticle wax extraction and analysis by gas chromatography was initially developed by CW Scherer [2]. This methodology was scaled up 10-fold, using 10 lice per sample (instead of 1 louse) and 80 μL extraction solvent instead of 8 μL. Work was conducted at En-Cas Analytical Laboratories of Winston-Salem, North Carolina, USA.

## Materials

Human head lice (*Pediculus humanus capitis*) used in this work were received as untreated live head lice collected from infested patients and shipped the day of collection at room temperature conditions. Head lice were stored refrigerated for several hours prior to testing.

Three different extraction solvents were used:

 • 50/50 w/w isopropyl myristate/decamethylcyclopentasiloxane (cyclomethicone D5) – (IPM/D5)-subject product

 • Iso-octane – a laboratory determined optimal extraction solvent for hydrocarbons

 • 50/50 methanol/water - control

### Sample preparation

Ten head lice (per sample) were transferred individually with tweezers to clean empty 350-μL glass GC vial inserts. Specimens were covered with 80 μL of any of the three extraction solvents then allowed to incubate at room temperature for 10 minutes. The samples were then mixed by aspiration. Solvent samples containing the extracted lice cuticle hydrocarbons (CHCs) were transferred to a new unused insert contained in a 2-mL crimp top vial which was subsequently sealed and held for later analysis.

### Gas chromatography

A gas chromatograph equipped with a flame ionization detector (GC/FID) was used to determine the presence of hydrocarbons in the three head lice extracts. A standard containing a series of known hydrocarbons (C21-40) was used for qualitative sample comparison.

## Results

In his dissertation, Scherer reported four specific hydrocarbons present in significant amounts in every head louse sample he studied [2]. These four hydrocarbons represent approximately 50% of the cuticle hydrocarbons of head lice, and had retention time indices (RI) of 2500, 2700, 2900 and 3075.

The peaks that corresponded to the four retention time indices attributed to the head louse by Scherer were integrated and compared qualitatively to the alkane standard.

The alkane calibration mixture, when run by GC, produced a chromatogram with regular peaks correlating with each respective component. The retention time indices for the four specific hydrocarbons of interest were 2500, 2700, 2900 and 3075.

The baseline experiment, comparing the chromatogram of the scaled-up iso-octane extraction (10 lice in 80 μL iso-octane) correlated very well with the analogous chromatogram published by Scherer. As expected, the main peaks observed for this sample had retention time indices of 2500, 2700, 2900 and 3075.

In a control experiment, 10 head lice were co-extracted by immersion with 50/50 methanol: water. This 50/50 methanol: water solution was found visually to provide entire wetting of the head louse. However, the chromatogram produced from the analysis of the aqueous methanol extract showed a complete lack of CHC removal efficiency.

**Figure 1:**
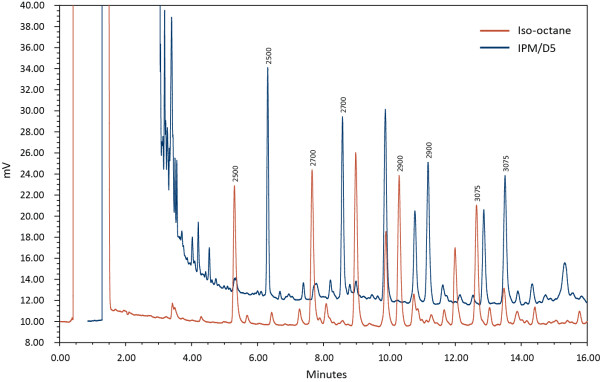
Gas Chromatogram demonstrating head lice epicuticle hydrocarbon extraction efficiency of isopropyl myristate/cyclomethicone D5 vs. iso-octane (offset for visualization purposes).

As seen in the gas chromatogram overlay (Figure 1), Isopropyl myristate/cyclomethicone D5 (IPM/D5) had equivalent efficacy extracting the four target hydrocarbons from the lice cuticular wax as iso-octane. The IPM/D5 (isopropyl myristate/cyclomethicone D5) (red) chromatogram is artificially shifted to the right, offset for visualization purposes, because the four peaks of interest were virtually identical in peak shape and height to those in the iso-octane chromatogram.

## Discussion

The study reported here was conducted to investigate the mechanism of action of IPM/D5, focusing on the potential role of IPM/D5 in physically disrupting the integrity of the head louse body’s outermost protection against dehydration. The cuticular wax that covers the exoskeleton of insects is vital in protecting them against dehydration and death. The wax layer of the epicuticle (outer layer) is composed of a matrix of hydrocarbons, and four specific hydrocarbons identified to be consistent among head lice by Scherer were selected for study in this work.

Isopropyl myristate/decamethylcyclopentasiloxane (IPM/D5) is a colorless, odorless solution. It was theorized that the mechanism of action for this solution involved disruption of the protective waxy coating covering the louse exoskeleton, leading to uncontrollable dehydration, and death.

It is known that suffocation of head lice is a slow process and can take up to 8 hours or more. Isopropyl myristate/cyclomethicone D5 has been shown clinically to have an 82% cure rate after two 10-minute treatments (one week apart) and therefore, suffocation is not a likely mechanism of action.

In this study gas chromatography was used to compare the hydrocarbon extraction efficiency of isopropyl myristate/cyclomethicone D5 with that of iso-octane as a control reference. Hydrocarbons specific to the waxy layer of the epicuticle of head lice were evaluated. This study showed that isopropyl myristate/cyclomethicone D5 effectively extracted the target hydrocarbons. Therefore, we deduce that the fast acting mechanism of action seen with IPM/D5 is via epicuticular hydrocarbon extraction, resulting in disruption of the waxy layer of the cuticle, followed by rapid water loss and death by dehydration.

### Overcoming resistance

These data support why a treatment with a physical mode of action, such as isopropyl myristate/cyclomethicone D5, can be comparable to a traditional pesticide. Pesticides, however, as evidenced in recent clinical trials, are increasingly facing issues of resistance globally. Burgess reported from a UK clinical study investigating IPM/D5 vs. 1% permethrin that IPM/D5 had 82% cure rate whereas the permethrin had only a 19% cure rate [6]. The development of resistance to pesticides such as permethrin is well documented [3].

## Conclusions

Isopropyl myristate/cyclomethicone D5 (IPM/D5) extracted the same specific cuticular wax hydrocarbons as those identified by Scherer to be critical to the survival of the head louse, therefore it can be deduced that IPM/D5 acts by a physical mode of action to kill head lice via disruption of the protective wax coating resulting in rapid dehydration. These data, combined with clinical study data support an explanation of the mechanism of action by dehydration, not suffocation.

Isopropyl myristate/cyclomethicone D5 is a colorless, odorless formulation that kills human head lice within 10 minutes by employing a physical mechanism of action that would not likely be subject to resistance. This formulation could be considered as a viable alternative to conventional pesticides.

## Abbreviations

DDT: Dichlorodiphenyltrichloroethane; FDA: Food and Drug Administration; GC: Gas Chromatograph; GC/FID: Gas Chromatograph equipped with a Flame Ionization Detector; IPM/D5: Isopropyl Myristate/cyclomethicone D5; MHRA: Medicines and Healthcare products Regulatory Agency; RI: Retention Index; w/w: By Weight (to describe concentration in solution).

## Competing interests

Piedmont Pharmaceuticals sponsored the study. Eric Barnett (EB) and Bert Clayton (BC) are employees of Piedmont Pharmaceuticals, the company that developed the non-pesticidal human head lice treatment composed of Isopropyl myristate/cyclomethicone D5 discussed in this paper. Kathleen Palma (KP) is a paid consultant of Piedmont Pharmaceuticals. This solution is marketed as Full Marks™ Solution by Reckitt Benckiser in certain European countries, Australia and Russia; as Resultz® by Medical Futures in Canada, Takeda in Belgium, and Lapidot in Israel. Tim Ballard (TB) of En-Cas Analytical Laboratories was paid on a fee for service basis to conduct the laboratory work, and does not have a competing interest.

## Authors’ contributions

KP designed the study and gave final approval to the publication. EB managed the overall research project and edited the publication. BC supervised the technical analysis. TB carried out the technical analysis. All authors read and approved the final manuscript.

## Authors’ information

Kathleen Palma is a PhD entomologist with over 20 years of experience in developing parasitology medicines for the pharmaceutical industry. Dr Palma is a co-inventor of the subject product.

## Pre-publication history

The pre-publication history for this paper can be accessed here:

http://www.biomedcentral.com/1471-5945/12/15/prepub
